# Postpartum family planning education during prenatal care: a cross-sectional study using descriptive statistics in Barrero, Dominican Republic

**DOI:** 10.3389/fgwh.2026.1804322

**Published:** 2026-07-22

**Authors:** Alexandra Van Cleave, Olivia Anne Foley, April Mabie, Caron Gray, Jason Beste, Michelle María Jiménez de Tavárez

**Affiliations:** 1 Arrupe Global Scholars & Partnership Program, Creighton University School of Medicine, Omaha, NE, United States; 2 Department of Obstetrics and Gynecology, Creighton University School of Medicine, Omaha, NE, United States; 3 Arrupe Global Scholars & Partnership Program, Creighton University School of Medicine, Phoenix, AZ, United States; 4 Facultad de Ciencias de la Salud, Pontificia Universidad Católica Madre y Maestra, Santiago de los Caballeros, Dominican Republic

**Keywords:** contraception, Dominican Republic, global health, insurance status, prenatal care

## Abstract

**Introduction:**

Barrero, Dominican Republic is a globally underrepresented area in research as no prior studies have been completed regarding women's health in this community. One important area of women's health is prenatal care as education is delivered to women that can impact the rest of their lives. The goal of this research project was to describe postpartum family planning information received during prenatal care for women in Barrero, Dominican Republic and create a database for this community regarding the prenatal care experience.

**Methods:**

A cross-sectional study consisting of a 28-question subset was administered in Barrero, a semi-rural, semi-urban community. 121 mothers, aged 18–50, with children 0–12 years old living in Barrero were surveyed in February 2025. Descriptive rates were reported as frequency (%).

**Results:**

Of the 216 pregnancies reported by 121 surveyed mothers, a postpartum family planning discussion occurred for 82% of women during prenatal care. Notably, of 92 women using contraception at the time of survey administration, 43.5% utilized tubal ligation as their primary method to avoid pregnancy. Other methods reported included oral contraception (21.7%), arm implant (17.4%), injections (15.2%), and only 1.1% reported using condoms.

**Conclusion:**

Comprehensive knowledge regarding contraception options is essential for women to make a personalized family planning decision. Future research projects should investigate if causal relationship exists between postpartum family planning education discussions and future contraception utilization. These projects could then shape provider education regarding postpartum family planning conversations.

## 1 Introduction

According to the World Health Organization (WHO) in 2023, over 700 women worldwide died daily from preventable causes related to pregnancy and childbirth (1). Prenatal care, defined as the care provided while a person is pregnant, aims to improve perinatal outcomes and support women throughout pregnancy, childbirth and postpartum life (2). Integral aspects of prenatal counseling include both family planning and contraception education, as both topics directly impact the health of mothers and fetuses and allow women the ability to make choices regarding their healthcare (3).

The WHO defines family planning education as information that gives people the choice of how many children they have and the time between pregnancies (4). Family planning includes both pregnancy prevention conversations as well as management and treatment of infertility, both of which provide space and information for women to make autonomous choices (5). Additionally, family planning education with every pregnancy is essential as each pregnancy carries unique risks and adequate spacing between pregnancies can significantly improve a mother's wellbeing (6). Globally, there are 1.1 billion women of reproductive age who need family planning (7). Of these, 874 million utilize some form of modern contraception, while 164 million still lack contraception (5, 7). The women lacking contraception describe limited availability of contraceptive choices, worsened if providers have a bias against certain methods (7). Women must receive education regarding contraceptive methods to decide if they want to utilize them, and prenatal visits can increase this awareness of the variety of contraceptive options. Previous work has demonstrated that prenatal counseling and education is a significant predictor of post-birth contraceptive use, allowing women the ability to decide if they want future pregnancies and how far apart they are (8).

Spacing out pregnancies by increasing the interpregnancy interval, if that aligns with a patient's preference, may decrease maternal mortality rates. Short interpregnancy intervals are linked to increased maternal mortality ratios, especially related to an increased risk of postpartum hemorrhage (9). Of note, women's health data presented from UNICEF showed that in the Dominican Republic (DR), 93% of women aged 15–49 years attend at least 4 prenatal care visits during pregnancy (10). Also, a systematic review establishes that prenatal care is associated with reduced maternal mortality (11). Paradoxically, 107 women die per every 100,000 live births, which is much higher than the Latin American & Caribbean regional average of 77 maternal deaths per 100,000 live births, demonstrating something may be missing from prenatal care (12, 13). A retrospective cohort study of 176,092 pregnancies in the United States documented a wide range of receipt of guideline-based care for eight selected American College of Obstetrics and Gynecologists guidelines (14). Therefore, this demonstrates prenatal care inconsistency and family planning education may differ greatly between patients affecting the ability of the patient to choose if and when they desire pregnancy prevention. Without clear direction of interpregnancy interval, risk of maternal hemorrhage increases impacting maternal mortality rates.

Studies conducted within the DR demonstrate widespread usage of contraception, yet limited knowledge exists regarding contraception methods. A recent study from 2022 interviewing adolescents attending university found that 25% of adolescents had knowledge about condoms and 23% knew about the oral anti-contraceptive pill method (15). A different study interviewed Dominican women attending a specific primary clinic and found that only 52.3% knew about hormonal birth control methods (16). Even with limited knowledge about contraceptive options, contraception usage in the DR is 71.8%, representing one of the highest rates among Latin American and Caribbean countries (17, 18). Of sexually active women utilizing a contraceptive method in the DR, approximately 55% are using permanent contraception (tubal ligation) (19). When one form of contraception is being used by over 50% of the population, it is known as a skewed method mix and demonstrates a lack of alternative methods or biased options delivered from their healthcare provider (20). This prior data does not elucidate the impact of insurance status on the contraceptive methods chosen, nor does it include subset data on specific regions of the DR.

Both public insurance and private insurance are offered in the DR. However, those with financial means prefer private hospitals secondary to a perceived higher quality care (21, 22). The public clinics and hospitals that provide free medical treatment are often poorly equipped and understaffed (21). Ladak et al. demonstrated in an umbrella review, including studies about 20 countries, that education provided at prenatal visits, including information regarding family planning, is greatly impacted by insurance systems (23). The aim of this study was to describe the information received during prenatal care about postpartum family planning between patients who had private, public, and no insurance in Barrero, DR along with other demographic factors regarding contraception utilization.

This research provides a descriptive analysis of a community that has never been researched, Barrero DR. In unstudied regions, representation with descriptive studies can help better understand the status of contraceptive care and postpartum family planning discussions in semi-urban settings in the DR. Through the creation of a community database, future research can build from this starting point to better elucidate any potential causal relationship between postpartum family planning discussions during prenatal care and future contraception usage.

## 2 Materials and methods

This descriptive, cross-sectional study aimed to evaluate the association of insurance status on family planning and contraception information provided during prenatal care appointments for women living in the semi-rural, semi-urban community of Barrero, DR. The study population included mothers with children 0–12 years old at the time of data collection. Previous surveys conducted by a partner organization, Centro de Educación para la Salud Integral (CESI)/Misión ILAC, a Dominican nonprofit, estimate the population of Barrero to be around 800 families. The goal sample size was 10% of the population or 80 women. Although no power calculation was utilized, this number was selected by a senior author on the paper as a goal convenience sample size. No previous data or estimates exist outlining the projected number of families that fit this study's inclusion criteria in the community; therefore, the goal sample size was set by the faculty of this project. The research team partnered with the Barrero community, Misión ILAC, and Pontificia Universidad Cátolica Madre y Maestra (PUCMM) to complete this project. Faculty at PUCMM acted as a primary support for the development of this project, survey, and data collection.

This survey was developed by a team of maternal health experts from the DR, including previously utilized surveys as a basis (8, 16, 24). The survey was then edited by senior health professionals on the team. The 76-question survey, written originally in English, included the following categories within the 28-question subset: demographic information, prenatal care experience, postpartum family planning education received during prenatal care, and current contraceptive use. This subset was pre-specified prior to data collection. The survey was translated into Spanish utilizing a professional translation service and reviewed for cultural accuracy by MJ. The Spanish survey and an English translation can be found in Supplementary Figures 1, 2: the subset of questions utilized for this project were 1–20 and 69–76.

Prior to data collection, a map of Barrero was created by the authors with the assistance of community members living there. The community was divided into ten zones to assure community coverage. See Supplementary Figure 3. AV, OF, and AM walked through the community to add details to the map and increase their familiarity of the community.

During data collection, AV, OF, and AM were led by community members to areas with children aged 0–12 years old. Teams relied in part on community members' knowledge of the community to determine location of possible participants within each zone. Data was gathered utilizing a paper-based quantitative survey via house-to-house sampling during February 2025. Women from each zone were surveyed to ensure complete representation of the Barrero community. Representation was assessed through at least one participant from each zone, while within zones, convenience sampling was utilized according to community members' knowledge. Researchers orally asked survey questions and recorded responses in the respective category. Exact answers were written down if the “other” category was chosen. All surveys were conducted in Spanish by authors AV, OF, and AM.

Individual consent was obtained from every participant via an informed consent form prior to administration of the survey. Women were included if they had at least one child aged 0–12 years and were age 18–61 at the time of survey (25). Participants who did not receive prenatal care in the DR and/or did not give birth in the DR were excluded.

Upon completion of data collection, responses were coded in a password-protected Excel spreadsheet. Descriptive rates were reported as frequency (%).

## 3 Results

Overall, 121 women were interviewed. Of these women, 110 identified as Dominican, 10 identified as Haitian and one woman preferred not to answer. Ten women identified Creole as their native language, while 111 identified Spanish as their native language. Women ranged from 18 to 50 years old with number of pregnancies per each participant ranging from one to thirteen. The women interviewed discussed 236 pregnancies. Data about sexual health education was only collected for 216 pregnancies, as some women preferred not to discuss this information and others lacked sufficient time to complete the survey.

**Figure 1:**
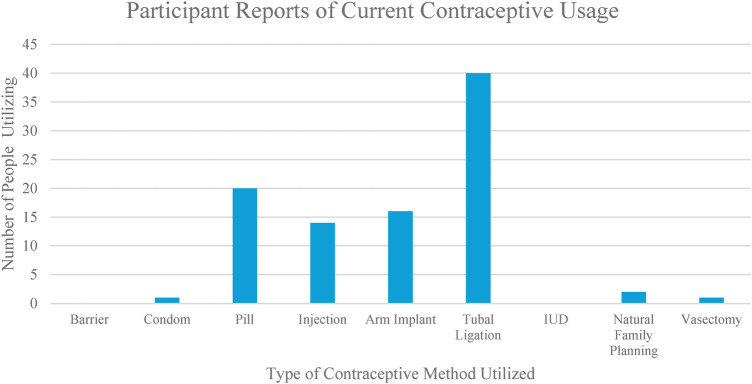
Type of contraceptive method utilized. This bar graph demonstrates the type of contraceptive method utilized by participants at the time of survey administration.

Of the 121 women interviewed, 114 women responded about current desire for more children or not, with 30 responding they did (26.31% of respondents). Beyond that, 92 women of 119 women who answered this question stated that they currently were using a contraceptive method with Figure 1 demonstrating the type of contraception method utilized. Two of these women reported utilizing multiple methods (one reported both the pill and natural family planning; one reported both tubal ligation and vasectomy). The distribution of contraceptive usage by demographic data found that the most utilized contraceptive method was tubal ligation with overlapping standard deviations between ages and no trends identified between insurance types as seen in Tables 1, 2. Table 1 demonstrates the number of women reporting utilizing each form of contraception as well as the calculated percentage per method. Multiple women reported using several forms of contraception. These results demonstrate that the most widely utilized method was tubal ligation at 43.5% with oral contraception, the pill second at 21.7%. The average age for these two types of contraception trended older at 29.46 years old and 25.15 years old respectively. For insurance status, those utilizing the pill and the arm implant primarily had private insurance (70% and 60% of women utilizing these methods respectively). However, for the injection, 50% of women did not have any form of medical insurance. Receiving prenatal education about postpartum family planning was not impacted by insurance status as seen in Table 3 with 82% of respondents reporting that they discussed postpartum family planning during prenatal care. Finally, data on ease of access to contraception, regardless of current usage, was collected, and most women endorsed ease accessing contraception if desired with around 90% endorsing accessibility if desired. Various demographic factors considered to potentially influence access were analyzed including current employment status, highest education level achieved, current insurance status as well as nationality and trends can be seen in Table 4. Of these factors, none appear to impact ease of access to contraceptive method. The question's phrasing can be seen in Supplementary Figures 1, 2 in question 76.

**Table 1:** Contraceptive method utilized and average Age per method.

Contraceptive method	# of women utilizing	% of women utilizing*	Average age, *y* (SD)
Barrier	0	0	—
Condom	1	1.09	25.00 (-)
Pill	20	21.74	25.15 (5.04)
Injection	14	15.22	24.98 (6.77)
Arm Implant	16	17.39	21.08 (5.80)
Tubal Ligation	40	43.48	29.46 (5.40)
IUD	0	0	—
Natural Family Planning	2	2.17	19.50 (-)
Vasectomy	1	1.09	30.00 (-)

*These numbers were calculated among women who responded utilizing at least one form of contraception. There were several women who reported multiple forms of contraception.

**Table 2:** Contraceptive method utilized and insurance status per method.

Contraceptive method	# of Women utilizing*	% of women without insurance	% of women with public insurance	% of women with private insurance
Barrier	0			
Condom	1	0	0	100
Pill	20	25	5	70
Injection	14	50	21	29
Arm implant	16	27	13	60
Tubal ligation	40	20	34	46
IUD	0			
Natural family Planning	2	50	50	0
Vasectomy	1	0	0	100

*Current insurance status was missing for one respondent.

**Table 3:** Family planning education based on insurance status during prenatal care per pregnancy.

Education received about family planning during prenatal care per pregnancy				
Prenatal care insurance status	People Reporting “Yes”		People Reporting “No”	
None	54	25.6%	11	5.2%
Public	26	12.3%	11	5.2%
Private	93	44.1%	16	7.6%

**Table 4:** Ease of access to a contraceptive method based on a variety of demographic factors.

Ease of access to contraceptive method					
Demographic Factors		People Reporting “Yes”		People Reporting “No”	
Age, *y* (SD)		26 (6)		25 (5)	
Current employment status	Unemployed	55	48.7%	4	3.5%
	Formal Employment	25	22.1%	3	2.7%
	Informal Employment	22	19.5%	4	3.5%
Highest level of education attained	None	3	2.7%	1	0.9%
	Primary	32	28.3%	5	4.4%
	Secondary	51	45.1%	4	3.5%
	University	16	14.2%	1	0.9%
Current insurance status	None	28	26.4%	3	2.8%
	Public	20	18.9%	1	0.9%
	Private	48	45.3%	6	5.7%
Nationality	Dominican	94	83.9%	9	8.0%
	Haitian	7	6.3%	2	1.8%

## 4 Discussion

### 4.1 Key findings

This study described postpartum family planning education during prenatal care and contraceptive method utilization for women living in Barrero, DR. The results demonstrated that most women interviewed received information regarding postpartum family planning during prenatal care. In addition, almost all women surveyed stated it is or would be easy to access contraception if they desired it. Finally, most women surveyed who were utilizing a contraceptive method underwent a tubal ligation.

Throughout this study, the conversations that occur during prenatal care were evaluated for the presence of family planning education information. However, the content and quality of these conversations is outside the scope of this study. Many women described experiencing something called a “charla” during prenatal care. A charla consists of a group attending a short informal session where a community health worker delivers knowledge regarding a specific topic (26). These sessions, when offered during prenatal care, build a community among the pregnant women which increases knowledge of the topic and can foster support throughout the group. Prior studies demonstrate that knowledge about prenatal vitamin utilization significantly increased after attending a charla and an organization called “La Charla” demonstrated increased sexual health knowledge after a charla (26, 27).

The data on contraceptive methods utilized among the women of Barrero highlighted an interesting trend, as 43% of woman using contraception reported tubal ligation was their form of birth control. Previous studies in the DR indicated more than 50% of women utilized permanent sterilization as their contraceptive method (28). A “skewed contraceptive method” occurs when more than 50% of women choose a single method, and this pattern may be secondary to insufficient availability of options or intimidation to use a certain method (28). Forty-three percent is not a majority; however, it is much higher than the next highest percentage of 21.7% for the oral contraceptive pill, even though it is less than the national data (28). Although not meeting the criteria for a skewed contraceptive method mix, these results may indicate a lack of contraception options or inadequate education. Although not a part of the survey questions, several women mentioned that their tubal ligation was not their choice. These were spontaneous comments and thus, are only an anecdotal finding. Whether the procedure was performed secondary to an emergency, or for another reason, is outside the scope of this study. However, it identifies a need for further investigation into operations performed without consent that have permanent impacts on women as a hypothesis-generating observation.

Another interesting finding was that only one woman interviewed endorsed the utilization of condoms. Condoms are an effective method for both preventing pregnancy and decreasing STI transmission (29). Prior studies have demonstrated that economically disadvantaged women utilize condoms less often (30). Future investigations could identify why the utilization of this form of contraception is so limited in this community and if their socioeconomic status is influencing these women's choice of contraception.

Finally, this project has created a database describing the current utilization of contraception and the prevalence of postpartum family planning conversations. These conversations can greatly impact a women's health after pregnancy and are an important intervention. This database can be utilized as a starting point for future research projects as it represents an understudied community and contributes to increased awareness of women's health for these women. It also improves the representation of the DR because they have been historically underrepresented within global published research.

### 4.2 Limitations

Barrero is a semi-urban community and may not be representative of maternal health in other regions of the country. Although focusing on a single community limits the generalizability of the findings, this study provides a locally grounded assessment that responds to a need identified by Misión ILAC and generates exploratory evidence to inform community intervention programs. The study sample was non-probabilistic due to the specific eligibility criteria of the population of interest. However, a household-based quota approach was applied to maximize zonal coverage within Barrero. While house-to-house mapping may introduce a bias, it also contributes to filling a gap in the literature on community-based research. Furthermore, the study adds value by demonstrating how first-hand knowledge from community members can be leveraged to support data collection.

This study also may suffer from recall bias, as the population consists of mothers with children 0–12 years of age who were asked about their prenatal care experiences for all pregnancies. Some women may have experienced differential recall by parity and the outcome of the pregnancy; for example, a pregnancy ending in an emergency C-section may be better remembered than one which ended in a normal spontaneous vaginal delivery. This differential recall bias may impact some of the responses given for survey questions. However multiple studies showed that maternal recollection of events such as a birth is fairly strong, so it is reasonable to believe this study's findings may be accurate (31, 32). Finally, this survey was administered in Spanish by non-Native speakers. Although surveys were translated using professional services and edited by native Dominicans, there may have been some cultural differences and misunderstandings during data collection leading to a possible miscommunication or social desirability bias. Moreover, seven women who identified Creole as their native language relied on interpretation between Spanish to Creole by other community members, limiting direct understanding of the answers provided by participants. No back-translation or quality checks were utilized for these seven surveyed participants who utilized community interpreters to complete their surveys. Women who only spoke Creole, and lacked a willing and able interpreter, were excluded from the study.

### 4.3 Future investigations

Women in Barrero, Dominican Republic do not currently have a standardized source of sexual health education. Variable education about postpartum family planning education exists during prenatal care visits, and while many contraceptive options are utilized, tubal ligation remains the most common. Limited diversity in contraception method may indicate limited knowledge or access regarding contraceptive options with many women endorsing a tubal ligation. Future studies should investigate the content delivery of postpartum family planning information, including which options are discussed and how risks/benefits are described. Finally, a future analysis should elaborate on motivations for and circumstances surrounding tubal ligation procedures.

## Data Availability

The raw data supporting the conclusions of this article will be made available by the authors, without undue reservation.

## Supplementary material

Supplementary Figure 1 Demonstrates the Spanish version of the full 76-question survey.

Supplementary Figure 2 Demonstrates the English version of the full 76-question survey.

Supplementary Figure 3 Identifies the ten zones of the community and shows how the community was mapped.
