# Supplementary material for: Postpartum family planning education during prenatal care: a cross-sectional study using descriptive statistics in Barrero, Dominican Republic
Source: Front Glob Womens Health. 2026 Jul 22;7:1804322. doi: 10.3389/fgwh.2026.1804322 (PMC13437963; doi:10.3389/fgwh.2026.1804322)
Supplement: Supplementary file 1 [file Datasheet1.pdf]

PONTIFICIA UNIVERSIDAD CATÓLICA MADRE Y MAESTRA
FACULTAD DE CIENCIAS DE LA SALUD
ESCUELA DE MEDICINA

RELATIONSHIP BETWEEN INSURANCE STATUS AND PRENATAL CARE IN CESI/MISIÓN ILAC CAMPOS IN THE DOMINICAN REPUBLIC

Por: *Foley, Olivia; Mabie, April; Van Cleave, Alexandra*
Asesoras oficiales: *Dra. Michelle Jiménez de Tavárez, MD, PhD; Dra. Caron Gray, MD*
Asesores metodológicos*: Dr. Jason Beste, MD, MPH; Dr. Ryan Walters, PhD*

Nombre del participante: ______________________ Apellido: _________________________ Número de identificación: ____________

**Datos demográficos:**	
1. ¿Cuántos años tiene?	Años: ________
2. ¿Usted tiene seguro médico ahora?	Sí<br>No<br>No recuerdo
3. ¿Si tiene seguro médico, qué tipo de seguro tiene actualmente?	☐ Público (SeNaSa)<br>☐ Privado<br>☐ Ninguno
4. ¿Me podría mostrar su tarjeta?	Sí<br>No<br>No la tengo<br>Prefiero no mostrarla
5. ¿Qué tipo de empleo tiene actualmente?	☐ Empleo formal<br>☐ Empleo informal<br>☐ Sin empleo
6. ¿Cuál es el nivel de estudios más alto que alcanzó?	☐ Ninguno<br>☐ Primaria<br>☐ Secundaria<br>☐ Universidad
7. ¿En qué fecha fue el primer día de su último período menstrual?	Día/mes/año: _________<br>Menopausia
8. ¿Está embarazada actualmente?	☐ Sí

	☐ No
9. ¿Cuántos embarazos tuvo?	Total: ____________
10. ¿Cuál es su nacionalidad?	☐ Dominicana<br>☐ Haitiana<br>☐ Otra: __________<br>☐ Prefiero no responder

11. ¿Cuál es su lengua materna?	☐ Español<br>☐ Criollo<br>☐ Inglés<br>☐ Otra: ___________<br>☐ Prefiero no responder			
12. ¿Habló alguien en su lengua materna durante el proceso de parto de sus hijos?	☐ Sí<br>☐ No			
13. ¿Cuáles son las edades actuales y los nombres de sus hijos? Los datos de identificación se destruirán después de la entrevista.	Niño 1: ____________	Niño 2: __________	Niño 3: ____________	Niño 4: ____________
**<u>Atención prenatal:</u>**				
Estas preguntas se formularán para cada embarazo. Responda a las siguientes preguntas sobre su experiencia de atención prenatal con __________ (indique el nombre del niño).	Niño A: __________________	Niño B: __________________	Niño C: __________________	Niño D: __________________
14. En su embarazo de ______ (indique aquí el nombre del niño), ¿recibió atención prenatal?	☐ Sí<br>☐ No	☐ Sí<br>☐ No	☐ Sí<br>☐ No	☐ Sí<br>☐ No
15. En su embarazo de ______ (indique aquí el nombre del niño), ¿usted necesitó de un seguro médico para sus chequeos prenatales?	Sí<br>No<br>No recuerdo<br>No tenía seguro	Sí<br>No<br>No recuerdo<br>No tenía seguro	Sí<br>No<br>No recuerdo<br>No tenía seguro	Sí<br>No<br>No recuerdo<br>No tenía seguro
16. ¿Qué tipo de seguro tenía durante este embarazo de _____ (indique aquí el nombre del niño)?	Privado<br>Público<br>No recuerdo<br>No tenía seguro	Privado<br>Público<br>No recuerdo<br>No tenía seguro	Privado<br>Público<br>No recuerdo<br>No tenía seguro	Privado<br>Público<br>No recuerdo<br>No tenía seguro
Si la respuesta es **negativa para 14**, continúe con la pregunta 27.<br>Si la respuesta es **afirmativa**, continúe con la pregunta 17.				
17. ¿Dónde recibió la atención?	☐ Hospital público<br>☐ Hospital privado<br>☐ En casa<br>☐ En mi comunidad<br>☐ Clínica comunitaria<br>☐ Otro: ___________	☐ Hospital público<br>☐ Hospital privado<br>☐ En casa<br>☐ En mi comunidad<br>☐ Clínica comunitaria<br>☐ Otro: ___________	☐ Hospital público<br>☐ Hospital privado<br>☐ En casa<br>☐ En mi comunidad<br>☐ Clínica comunitaria<br>☐ Otro: ___________	☐ Hospital público<br>☐ Hospital privado<br>☐ En casa<br>☐ En mi comunidad<br>☐ Clínica comunitaria<br>☐ Otro: ___________
18. ¿Quién fue su principal proveedor de atención prenatal? (Elija una sola opción).	☐ Médico/a<br>☐ Prof. de enfermería<br>☐ Partera	☐ Médico/a<br>☐ Prof. de enfermería<br>☐ Partera	☐ Médico/a<br>☐ Prof. de enfermería<br>☐ Partera	☐ Médico/a<br>☐ Prof. de enfermería<br>☐ Partera

	☐ Miembro de la familia<br>☐ Cooperadora<br>☐ Profesional de ILAC<br>☐ Otro: ___________	☐ Miembro de la familia<br>☐ Cooperadora<br>☐ Profesional de ILAC<br>☐ Otro: ___________	☐ Miembro de la familia<br>☐ Cooperadora<br>☐ Profesional de ILAC<br>☐ Otro: ___________	☐ Miembro de la familia<br>☐ Cooperadora<br>☐ Profesional de ILAC<br>☐ Otro: ___________
19. Aproximadamente, ¿cuántas consultas prenatales tuvo?	☐ 0<br>☐ 1 a 3<br>☐ 4 a 7<br>☐ 8 o más	☐ 0<br>☐ 1 a 3<br>☐ 4 a 7<br>☐ 8 o más	☐ 0<br>☐ 1 a 3<br>☐ 4 a 7<br>☐ 8 o más	☐ 0<br>☐ 1 a 3<br>☐ 4 a 7<br>☐ 8 o más

20. ¿En qué momento del embarazo comenzó a asistir a las consultas?	☐ De 0 a 3 meses<br>☐ De 4 a 6 meses<br>☐ >7 meses	☐ De 0 a 3 meses<br>☐ De 4 a 6 meses<br>☐ >7 meses	☐ De 0 a 3 meses<br>☐ De 4 a 6 meses<br>☐ >7 meses	☐ De 0 a 3 meses<br>☐ De 4 a 6 meses<br>☐ >7 meses
21. Durante las consultas, ¿alguien le habló sobre la alimentación de su hijo o la lactancia?	☐ Sí<br>☐ No	☐ Sí<br>☐ No	☐ Sí<br>☐ No	☐ Sí<br>☐ No
22. Durante las consultas, ¿alguien le habló sobre su plan de parto?	☐ Sí<br>☐ No	☐ Sí<br>☐ No	☐ Sí<br>☐ No	☐ Sí<br>☐ No
23. ¿Planeaba una cesárea o un parto vaginal?	☐ Vaginal<br>☐ Cesárea	☐ Vaginal<br>☐ Cesárea	☐ Vaginal<br>☐ Cesárea	☐ Vaginal<br>☐ Cesárea
24. En la mayoría de las ocasiones, ¿cómo acudía a las consultas?	☐ Automóvil<br>☐ Caminando<br>☐ Automóvil de otra persona<br>☐ Motocicleta<br>☐ Transporte público<br>☐ Guagua<br>☐ Animal<br>☐ Otro: ___________	☐ Automóvil<br>☐ Caminando<br>☐ Automóvil de otra persona<br>☐ Motocicleta<br>☐ Transporte público<br>☐ Guagua<br>☐ Animal<br>☐ Otro: ___________	☐ Automóvil<br>☐ Caminando<br>☐ Automóvil de otra persona<br>☐ Motocicleta<br>☐ Transporte público<br>☐ Guagua<br>☐ Animal<br>☐ Otro: ___________	☐ Automóvil<br>☐ Caminando<br>☐ Automóvil de otra persona<br>☐ Motocicleta<br>☐ Transporte público<br>☐ Guagua<br>☐ Animal<br>☐ Otro: ___________
25. ¿Fue alguien a las consultas prenatales con usted?	☐ Sí<br>☐ No	☐ Sí<br>☐ No	☐ Sí<br>☐ No	☐ Sí<br>☐ No
26. En caso afirmativo, ¿quién fue con usted? (Elija todas las opciones que correspondan).	☐ Partera<br>☐ Pareja<br>☐ Padre(s)<br>☐ Amigo(s)<br>☐ Otros hijos<br>☐ Otro: ___________	☐ Partera<br>☐ Pareja<br>☐ Padre(s)<br>☐ Amigo(s)<br>☐ Otros hijos<br>☐ Otro: ___________	☐ Partera<br>☐ Pareja<br>☐ Padre(s)<br>☐ Amigo(s)<br>☐ Otros hijos<br>☐ Otro: ___________	☐ Partera<br>☐ Pareja<br>☐ Padre(s)<br>☐ Amigo(s)<br>☐ Otros hijos<br>☐ Otro: ___________

Repita las preguntas de la 14 a la 26 para todos los embarazos y, luego, continúe con la pregunta 27.

Método de parto:

Estas preguntas se formularán para cada embarazo. Responda a las siguientes preguntas sobre su experiencia de parto con ___________ (indique el nombre del niño).	Niño _________________	Niño _________________	Niño _________________	Niño _________________
27. ¿Usted necesitó de un seguro médico para dar a luz?	Sí<br>No<br>No recuerdo	Sí<br>No<br>No recuerdo	Sí<br>No<br>No recuerdo	Sí<br>No<br>No recuerdo

	No la tenía	No la tenía	No la tenía	No la tenía
28. Si necesitó seguro médico para dar a luz, ¿qué tipo de seguro tenía para dar a luz?	Privado<br>Público<br>No recuerdo<br>No tenía seguro	Privado<br>Público<br>No recuerdo<br>No tenía seguro	Privado<br>Público<br>No recuerdo<br>No tenía seguro	Privado<br>Público<br>No recuerdo<br>No tenía segur
29. ¿Dónde tuvo lugar el parto? Específicamente, ¿en qué ciudad o provincia?	Ciudad: ___________________	Ciudad: ___________________	Ciudad: ___________________	Ciudad: ___________________

30. ¿En qué tipo de establecimiento dio a luz?	☐ Hospital privado<br>☐ Hospital público<br>☐ Clínica comunitaria<br>☐ Hogar<br>☐ Otro: ___________	☐ Hospital privado<br>☐ Hospital público<br>☐ Clínica comunitaria<br>☐ Hogar<br>☐ Otro: ___________	☐ Hospital privado<br>☐ Hospital público<br>☐ Clínica comunitaria<br>☐ Hogar<br>☐ Otro: ___________	☐ Hospital privado<br>☐ Hospital público<br>☐ Clínica comunitaria<br>☐ Hogar<br>☐ Otro: ___________
31. ¿Qué distancia en minutos/horas tuvo que recorrer para dar a luz?	Horas:<br>_________________	Horas:<br>_________________	Horas:<br>_________________	Horas:<br>_________________
32. ¿Qué tipo de parto tuvo?	☐ Vaginal<br>☐ Cesárea	☐ Vaginal<br>☐ Cesárea	☐ Vaginal<br>☐ Cesárea	☐ Vaginal<br>☐ Cesárea
Si la respuesta es **vaginal**, continúe con las preguntas **33 a 35** .<br>Si la respuesta es **cesárea**, continúe con las preguntas **36 a 40** .				
33. ¿Qué la motivó a tener un parto vaginal?	☐ Preferencia del proveedor<br>☐ Tiempo de recuperación<br>☐ Preferencia de la pareja<br>☐ Esfuerzo percibido<br>☐ Dolor percibido<br>☐ Mi preferencia<br>☐ Otro: ___________	☐ Preferencia del proveedor<br>☐ Tiempo de recuperación<br>☐ Preferencia de la pareja<br>☐ Esfuerzo percibido<br>☐ Dolor percibido<br>☐ Mi preferencia<br>☐ Otro: ___________	☐ Preferencia del proveedor<br>☐ Tiempo de recuperación<br>☐ Preferencia de la pareja<br>☐ Esfuerzo percibido<br>☐ Dolor percibido<br>☐ Mi preferencia<br>☐ Otro: ___________	☐ Preferencia del proveedor<br>☐ Tiempo de recuperación<br>☐ Preferencia de la pareja<br>☐ Esfuerzo percibido<br>☐ Dolor percibido<br>☐ Mi preferencia<br>☐ Otro: ___________
34. Si la respuesta a la pregunta **33 fue el tiempo de recuperación**, ¿cuánto duró su recuperación en semanas?	Semanas:<br>______________	Semanas:<br>______________	Semanas:<br>______________	Semanas:<br>______________
35. ¿Quién influyó más en su decisión de tener un parto vaginal?	☐ Yo misma<br>☐ Obstetra<br>☐ Prof. de enfermería<br>☐ Partera<br>☐ Pareja<br>☐ Padres<br>☐ Amigos<br>☐ Curso/educación<br>☐ Internet<br>☐ Televisión<br>☐ Redes sociales<br>☐ Periódico/revista/ libro/revista académica<br>☐ Otro: ___________	☐ Yo misma<br>☐ Obstetra<br>☐ Prof. de enfermería<br>☐ Partera<br>☐ Pareja<br>☐ Padres<br>☐ Amigos<br>☐ Curso/educación<br>☐ Internet<br>☐ Televisión<br>☐ Redes sociales<br>☐ Periódico/revista/ libro/revista académica<br>☐ Otro: ___________	☐ Yo misma<br>☐ Obstetra<br>☐ Prof. de enfermería<br>☐ Partera<br>☐ Pareja<br>☐ Padres<br>☐ Amigos<br>☐ Curso/educación<br>☐ Internet<br>☐ Televisión<br>☐ Redes sociales<br>☐ Periódico/revista/ libro/revista académica<br>☐ Otro: ___________	☐ Yo misma<br>☐ Obstetra<br>☐ Prof. de enfermería<br>☐ Partera<br>☐ Pareja<br>☐ Padres<br>☐ Amigos<br>☐ Curso/educación<br>☐ Internet<br>☐ Televisión<br>☐ Redes sociales<br>☐ Periódico/revista/ libro/revista académica<br>☐ Otro: ___________

36. ¿Le practicaron una cesárea de emergencia?	☐ Sí<br>☐ No	☐ Sí<br>☐ No	☐ Sí<br>☐ No	☐ Sí<br>☐ No
37. Si respondió que **no a la pregunta 36** , ¿la cesárea fue planificada?	☐ Sí<br>☐ No	☐ Sí<br>☐ No	☐ Sí<br>☐ No	☐ Sí<br>☐ No
38. ¿Qué motivó su decisión de someterse a una cesárea?	☐ Preferencia del proveedor<br>☐ Tiempo de recuperación<br>☐ Preferencia de la pareja<br>☐ Esfuerzo percibido<br>☐ Dolor percibido<br>☐ Mi preferencia<br>☐ Otro: ___________	☐ Preferencia del proveedor<br>☐ Tiempo de recuperación<br>☐ Preferencia de la pareja<br>☐ Esfuerzo percibido<br>☐ Dolor percibido<br>☐ Mi preferencia<br>☐ Otro: ___________	☐ Preferencia del proveedor<br>☐ Tiempo de recuperación<br>☐ Preferencia de la pareja<br>☐ Esfuerzo percibido<br>☐ Dolor percibido<br>☐ Mi preferencia<br>☐ Otro: ___________	☐ Preferencia del proveedor<br>☐ Tiempo de recuperación<br>☐ Preferencia de la pareja<br>☐ Esfuerzo percibido<br>☐ Dolor percibido<br>☐ Mi preferencia<br>☐ Otro: ___________
39. Si la respuesta a la pregunta **38 fue el tiempo de recuperación**, ¿cuánto duró su recuperación en semanas?	Semanas:<br>______________	Semanas:<br>______________	Semanas:<br>______________	Semanas:<br>______________
40. ¿Quién influyó más en su decisión de tener un parto por cesárea?	☐ Yo misma<br>☐ Obstetra<br>☐ Prof. de enfermería<br>☐ Partera<br>☐ Pareja<br>☐ Padres<br>☐ Amigos<br>☐ Curso/educación<br>☐ Internet<br>☐ Televisión<br>☐ Redes sociales<br>☐ Periódico/revista/ libro/revista académica<br>☐ Otro: ___________	☐ Yo misma<br>☐ Obstetra<br>☐ Prof. de enfermería<br>☐ Partera<br>☐ Pareja<br>☐ Padres<br>☐ Amigos<br>☐ Curso/educación<br>☐ Internet<br>☐ Televisión<br>☐ Redes sociales<br>☐ Periódico/revista/ libro/revista académica<br>☐ Otro: ___________	☐ Yo misma<br>☐ Obstetra<br>☐ Prof. de enfermería<br>☐ Partera<br>☐ Pareja<br>☐ Padres<br>☐ Amigos<br>☐ Curso/educación<br>☐ Internet<br>☐ Televisión<br>☐ Redes sociales<br>☐ Periódico/revista/ libro/revista académica<br>☐ Otro: ___________	☐ Yo misma<br>☐ Obstetra<br>☐ Prof. de enfermería<br>☐ Partera<br>☐ Pareja<br>☐ Padres<br>☐ Amigos<br>☐ Curso/educación<br>☐ Internet<br>☐ Televisión<br>☐ Redes sociales<br>☐ Periódico/revista/ libro/revista académica<br>☐ Otro: ___________
41. ¿Está satisfecha con su experiencia en el parto?	☐ Sí<br>☐ No	☐ Sí<br>☐ No	☐ Sí<br>☐ No	☐ Sí<br>☐ No

Repita las preguntas de la 27 a la 40 para todos los embarazos y, luego, continúe con la pregunta 42 .

Inmediatamente después del parto:

Estas preguntas se formularán para cada embarazo. Responda a las siguientes preguntas sobre su experiencia de atención posparto con __________ (indique el nombre del niño aquí).	Niño ________________	Niño ________________	Niño ________________	Niño ________________
42. ¿Tuvo contacto de piel con piel inmediatamente después del parto?	☐ Sí<br>☐ No	☐ Sí<br>☐ No	☐ Sí<br>☐ No	☐ Sí<br>☐ No
43. Inmediatamente después del nacimiento o después del contacto de piel con piel, ¿llevaron al bebé a otra habitación?	☐ Sí<br>☐ No	☐ Sí<br>☐ No	☐ Sí<br>☐ No	☐ Sí<br>☐ No

En caso **afirmativo**, continúe con la pregunta 44 .
En caso **negativo**, continúe con la pregunta 45 .

44. ¿Cuánto tiempo estuvieron separados?	☐ Menos de una hora<br>☐ De 1 a 2 horas<br>☐ >2 horas	☐ Menos de una hora<br>☐ De 1 a 2 horas<br>☐ >2 horas	☐ Menos de una hora<br>☐ De 1 a 2 horas<br>☐ >2 horas	☐ Menos de una hora<br>☐ De 1 a 2 horas<br>☐ >2 horas
45. ¿La animaron amamantar inmediatamente después del parto?	☐ Sí<br>☐ No	☐ Sí<br>☐ No	☐ Sí<br>☐ No	☐ Sí<br>☐ No
46. ¿La primera comida del bebé fue leche materna o fórmula?	☐ Leche materna<br>☐ Fórmula	☐ Leche materna<br>☐ Fórmula	☐ Leche materna<br>☐ Fórmula	☐ Leche materna<br>☐ Fórmula

Si la respuesta es **fórmula**, continúe con la pregunta 47
Si la respuesta es **leche materna**, comience a repetir las preguntas en la pregunta 42 para los embarazos restantes o continúe con la pregunta 48 .

47. ¿Quién le dio la fórmula?	☐ Médico/a<br>☐ Prof. de enfermería<br>☐ Partera<br>☐ Familia<br>☐ Amigos<br>☐ Yo misma<br>☐ Otro: ___________	☐ Médico/a<br>☐ Prof. de enfermería<br>☐ Partera<br>☐ Familia<br>☐ Amigos<br>☐ Yo misma<br>☐ Otro: ___________	☐ Médico/a<br>☐ Prof. de enfermería<br>☐ Partera<br>☐ Familia<br>☐ Amigos<br>☐ Yo misma<br>☐ Otro: ___________	☐ Médico/a<br>☐ Prof. de enfermería<br>☐ Partera<br>☐ Familia<br>☐ Amigos<br>☐ Yo misma<br>☐ Otro: ___________

Repita las preguntas de la 42 a la 47 para todos los embarazos y, luego, continúe con la pregunta 48 .

**Lactancia:**				
Estas preguntas se formularán para cada embarazo. Responda a las siguientes preguntas sobre su experiencia de atención posparto con __________ (indique el nombre del niño aquí).	Niño<br>________________	Niño<br>________________	Niño<br>________________	Niño<br>________________
48. ¿Alguna vez dio el pecho en exclusiva (lactancia materna exclusiva, LME), empezó con fórmula o empezó con una combinación de ambas?	☐ LME<br>☐ Fórmula<br>☐ Combinación	☐ LME<br>☐ Fórmula<br>☐ Combinación	☐ LME<br>☐ Fórmula<br>☐ Combinación	☐ LME<br>☐ Fórmula<br>☐ Combinación
Si la respuesta es **LME**, continúe con la pregunta 49 .<br>Si la respuesta es **fórmula**, continúe con la pregunta 50.<br>Si la respuesta es **una combinación**, continúe con la pregunta 51 .<br>Después de esa pregunta, continúe con la pregunta 52.				
49. ¿Por cuánto tiempo amamantó de forma exclusiva?	☐ Menos de 6 meses<br>☐ 6 meses<br>☐ De 7 a 12 meses<br>☐ >12 meses	☐ Menos de 6 meses<br>☐ 6 meses<br>☐ De 7 a 12 meses<br>☐ >12 meses	☐ Menos de 6 meses<br>☐ 6 meses<br>☐ De 7 a 12 meses<br>☐ >12 meses	☐ Menos de 6 meses<br>☐ 6 meses<br>☐ De 7 a 12 meses<br>☐ >12 meses
50. ¿Fue fácil conseguir fórmula?	☐ Sí<br>☐ No	☐ Sí<br>☐ No	☐ Sí<br>☐ No	☐ Sí<br>☐ No
51. Además de la leche materna, ¿qué otro alimento le dio a su bebé?	☐ Fórmula<br>☐ Leche animal/no humana<br>☐ Alimentos sólidos<br>☐ Otro: ______	☐ Fórmula<br>☐ Leche animal/no humana<br>☐ Alimentos sólidos<br>☐ Otro: ______	☐ Fórmula<br>☐ Leche animal/no humana<br>☐ Alimentos sólidos<br>☐ Otro: ______	☐ Fórmula<br>☐ Leche animal/no humana<br>☐ Alimentos sólidos<br>☐ Otro: ______
52. ¿Quién fue su principal influencia al momento de decidir cómo alimentar a su bebé? (Elija una sola opción).	☐ Médico/a<br>☐ Prof. de enfermería<br>☐ Partera<br>☐ Familia<br>☐ Vecino/a<br>☐ Redes sociales<br>☐ Libro<br>☐ Televisión<br>☐ Educación<br>☐ Otro: __________	☐ Médico/a<br>☐ Prof. de enfermería<br>☐ Partera<br>☐ Familia<br>☐ Vecino/a<br>☐ Redes sociales<br>☐ Libro<br>☐ Televisión<br>☐ Educación<br>☐ Otro: __________	☐ Médico/a<br>☐ Prof. de enfermería<br>☐ Partera<br>☐ Familia<br>☐ Vecino/a<br>☐ Redes sociales<br>☐ Libro<br>☐ Televisión<br>☐ Educación<br>☐ Otro: __________	☐ Médico/a<br>☐ Prof. de enfermería<br>☐ Partera<br>☐ Familia<br>☐ Vecino/a<br>☐ Redes sociales<br>☐ Libro<br>☐ Televisión<br>☐ Educación<br>☐ Otro: __________
Si la participante alimentó a su bebé con **LME o una combinación**, enuncie la pregunta 53A.<br>Si la participante alimentó a su bebé solo con **fórmula**, enuncie la pregunta 53B.				

53. A. ¿Sintió que recibió apoyo con la lactancia materna?	☐ Sí<br>☐ No	☐ Sí<br>☐ No	☐ Sí<br>☐ No	☐ Sí<br>☐ No
53. B. Si hubiera elegido amamantar, ¿cree que la habrían apoyado con esa elección?	☐ Sí<br>☐ No	☐ Sí<br>☐ No	☐ Sí<br>☐ No	☐ Sí<br>☐ No
Si la participante alimentó a su bebé con **LME o una combinación**, enuncie la pregunta 54A.<br>Si la participante alimentó a su bebé solo con **fórmula**, enuncie la pregunta 54B.				
54. A. Si tiene pareja, ¿la apoyó su pareja con la lactancia materna?	☐ Sí<br>☐ No<br>☐ No tengo pareja	☐ Sí<br>☐ No<br>☐ No tengo pareja	☐ Sí<br>☐ No<br>☐ No tengo pareja	☐ Sí<br>☐ No<br>☐ No tengo pareja
54. B. Si hubiera elegido amamantar y tiene pareja, ¿cree que su pareja la habría apoyado con esa elección?	☐ Sí<br>☐ No<br>☐ No tengo pareja	☐ Sí<br>☐ No<br>☐ No tengo pareja	☐ Sí<br>☐ No<br>☐ No tengo pareja	☐ Sí<br>☐ No<br>☐ No tengo pareja
Si la participante alimentó a su bebé con **LME o una combinación**, enuncie la pregunta 55A.<br>Si la participante alimentó a su bebé solo con **fórmula**, enuncie la pregunta 55B.				
55. A. Si tiene otros familiares, ¿la apoyaron sus otros familiares con la lactancia materna?	☐ Sí<br>☐ No<br>☐ No tengo otros familiares	☐ Sí<br>☐ No<br>☐ No tengo otros familiares	☐ Sí<br>☐ No<br>☐ No tengo otros familiares	☐ Sí<br>☐ No<br>☐ No tengo otros familiares
55 B. Si hubiera elegido amamantar y tiene otros familiares, ¿cree que algún otro familiar la habría apoyado en su elección de amamantar?	☐ Sí<br>☐ No<br>☐ No tengo otros familiares	☐ Sí<br>☐ No<br>☐ No tengo otros familiares	☐ Sí<br>☐ No<br>☐ No tengo otros familiares	☐ Sí<br>☐ No<br>☐ No tengo otros familiares
Repita las preguntas de la 48 a la 55 para todos los embarazos y, luego, continúe con la pregunta 56.				

Estas preguntas se formularán una sola vez:

56. ¿Cree que su leche materna contiene por sí sola suficientes nutrientes para alimentar a su bebé?	☐ Sí<br>☐ No
57. ¿Cree que produce suficiente leche materna para amamantar de forma exclusiva sin necesidad de suplementos?	☐ Sí<br>☐ No

58. ¿Hay algo específico que la ayudaría a amamantar o a sentirse más cómoda mientras amamanta? (Elija una sola opción).	☐ Más recursos educativos<br>☐ Mayor apoyo de los profesionales médicos inmediatamente después del parto<br>☐ Mayor apoyo familiar<br>☐ Mayor apoyo de los pares<br>☐ No necesito nada adicional<br>☐ Otro: ___________

Educación sexual:

Estas preguntas se formularán para cada embarazo. Responda a las siguientes preguntas sobre su experiencia de atención prenatal con ___________ (indique el nombre del niño aquí).	Niño ________________	Niño ________________	Niño ________________	Niño ________________
59. ¿Se realizó pruebas de infecciones de transmisión sexual (ITS) durante la atención prenatal?	☐ Sí<br>☐ No<br>☐ No recuerdo	☐ Sí<br>☐ No<br>☐ No recuerdo	☐ Sí<br>☐ No<br>☐ No recuerdo	☐ Sí<br>☐ No<br>☐ No recuerdo
60. ¿Recibió educación sobre las ITS durante la atención prenatal?	☐ Sí<br>☐ No<br>☐ No recuerdo	☐ Sí<br>☐ No<br>☐ No recuerdo	☐ Sí<br>☐ No<br>☐ No recuerdo	☐ Sí<br>☐ No<br>☐ No recuerdo

En caso **afirmativo o si no lo recuerda**, continúe con la pregunta 61.
En caso **negativo**, continúe con la pregunta 66.

61. ¿Recibió información sobre la gonorrea durante la atención prenatal?	☐ Sí<br>☐ No<br>☐ No recuerdo	☐ Sí<br>☐ No<br>☐ No recuerdo	☐ Sí<br>☐ No<br>☐ No recuerdo	☐ Sí<br>☐ No<br>☐ No recuerdo
62. ¿Recibió información sobre la clamidia durante la atención prenatal?	☐ Sí<br>☐ No<br>☐ No recuerdo	☐ Sí<br>☐ No<br>☐ No recuerdo	☐ Sí<br>☐ No<br>☐ No recuerdo	☐ Sí<br>☐ No<br>☐ No recuerdo
63. ¿Recibió información sobre la sífilis durante la atención prenatal?	☐ Sí<br>☐ No<br>☐ No recuerdo	☐ Sí<br>☐ No<br>☐ No recuerdo	☐ Sí<br>☐ No<br>☐ No recuerdo	☐ Sí<br>☐ No<br>☐ No recuerdo
64. ¿Recibió información sobre el VIH durante la atención prenatal?	☐ Sí<br>☐ No<br>☐ No recuerdo	☐ Sí<br>☐ No<br>☐ No recuerdo	☐ Sí<br>☐ No<br>☐ No recuerdo	☐ Sí<br>☐ No<br>☐ No recuerdo
65. ¿Recibió información sobre la hepatitis B durante la atención prenatal?	☐ Sí<br>☐ No<br>☐ No recuerdo	☐ Sí<br>☐ No<br>☐ No recuerdo	☐ Sí<br>☐ No<br>☐ No recuerdo	☐ Sí<br>☐ No<br>☐ No recuerdo

66. ¿Le diagnosticaron una ITS durante el embarazo?	☐ Sí<br>☐ No<br>☐ No recuerdo	☐ Sí<br>☐ No<br>☐ No recuerdo	☐ Sí<br>☐ No<br>☐ No recuerdo	☐ Sí<br>☐ No<br>☐ No recuerdo
En caso **afirmativo**, continúe con la pregunta 67.<br>En caso **negativo o si no recuerda,** continúe con la pregunta 69.				
67. ¿Con qué ITS? (Elija todas las opciones que correspondan).	☐ Gonorrea<br>☐ Clamidia<br>☐ Sífilis<br>☐ VIH<br>☐ Hepatitis B<br>☐ Otra: ___________	☐ Gonorrea<br>☐ Clamidia<br>☐ Sífilis<br>☐ VIH<br>☐ Hepatitis B<br>☐ Otra: ___________	☐ Gonorrea<br>☐ Clamidia<br>☐ Sífilis<br>☐ VIH<br>☐ Hepatitis B<br>☐ Otra: ___________	☐ Gonorrea<br>☐ Clamidia<br>☐ Sífilis<br>☐ VIH<br>☐ Hepatitis B<br>☐ Otra: ___________
68. ¿Recibió tratamiento para ello?	☐ Sí<br>☐ No<br>☐ No recuerdo	☐ Sí<br>☐ No<br>☐ No recuerdo	☐ Sí<br>☐ No<br>☐ No recuerdo	☐ Sí<br>☐ No<br>☐ No recuerdo
69. ¿Usó preservativos durante el embarazo?	☐ Sí<br>☐ No<br>☐ No recuerdo	☐ Sí<br>☐ No<br>☐ No recuerdo	☐ Sí<br>☐ No<br>☐ No recuerdo	☐ Sí<br>☐ No<br>☐ No recuerdo
En caso **afirmativo**, continúe con la pregunta 70.<br>En caso **negativo o si no recuerda,** continúe con la pregunta 72.				
70. ¿Con qué frecuencia usó preservativos durante el embarazo?	☐ Siempre<br>☐ A veces<br>☐ En pocas ocasiones	☐ Siempre<br>☐ A veces<br>☐ En pocas ocasiones	☐ Siempre<br>☐ A veces<br>☐ En pocas ocasiones	☐ Siempre<br>☐ A veces<br>☐ En pocas ocasiones
71. ¿Por qué los utilizó?	☐ Prevención de ITS<br>☐ Múltiples parejas<br>☐ Pareja nueva<br>☐ Diagnóstico positivo de ITS<br>☐ Otro: ___________	☐ Prevención de ITS<br>☐ Múltiples parejas<br>☐ Pareja nueva<br>☐ Diagnóstico positivo de ITS<br>☐ Otro: ___________	☐ Prevención de ITS<br>☐ Múltiples parejas<br>☐ Pareja nueva<br>☐ Diagnóstico positivo de ITS<br>☐ Otro: ___________	☐ Prevención de ITS<br>☐ Múltiples parejas<br>☐ Pareja nueva<br>☐ Diagnóstico positivo de ITS<br>☐ Otro: ___________
72. ¿Recibió educación sobre planificación familiar durante la atención prenatal?	☐ Sí<br>☐ No<br>☐ No recuerdo	☐ Sí<br>☐ No<br>☐ No recuerdo	☐ Sí<br>☐ No<br>☐ No recuerdo	☐ Sí<br>☐ No<br>☐ No recuerdo
Repita las preguntas de la 59 a la 72 para todos los embarazos y, luego, continúe con la pregunta 73.				

Estas preguntas se formularán una sola vez:

73. Actualmente, ¿desea tener más hijos?	☐ Sí<br>☐ No
74. ¿Usted o su(s) pareja(s) toman alguna medida o usan algún método para retrasar o evitar el embarazo?	☐ Sí<br>☐ No
En caso **afirmativo**, continúe con la pregunta 75.<br>En caso **negativo**, continúe con la pregunta 76.	
75. ¿Qué método(s) usan? (Elija todas las opciones que correspondan).	☐ Barrera<br>☐ Preservativo<br>☐ Píldora<br>☐ Inyección<br>☐ Implante en el brazo<br>☐ Ligadura de trompas<br>☐ DIU<br>☐ Planificación familiar natural/días estándares<br>☐ Vasectomía<br>☐ Otro: ___________
76. Ya sea que use un método de planificación familiar o no, ¿es fácil acceder a un método de planificación familiar?	☐ Sí<br>☐ No
