# Supplementary material for: Postpartum family planning education during prenatal care: a cross-sectional study using descriptive statistics in Barrero, Dominican Republic
Source: Front Glob Womens Health. 2026 Jul 22;7:1804322. doi: 10.3389/fgwh.2026.1804322 (PMC13437963; doi:10.3389/fgwh.2026.1804322)
Supplement: Supplementary file 2 [file Datasheet2.pdf]

PONTIFICIA UNIVERSIDAD CATÓLICA MADRE Y MAESTRA
FACULTAD DE CIENCIAS DE LA SALUD
ESCUELA DE MEDICINA

RELATIONSHIP BETWEEN INSURANCE STATUS AND PRENATAL CARE IN CESI/MISIÓN ILAC CAMPOS IN THE DOMINICAN REPUBLIC

By: *Foley, Olivia; Mabie, April; Van Cleave, Alexandra*
Asesoras Oficial: *Dra. Michelle Jiménez de Tavárez, MD, PhD; Dr. Caron Gray, MD*
Asesore Metodológicos*: Dr. Jason Beste, MD, MPH; Dr. Ryan Walters, PhD*

Participant First Name: ______________________ Last Name: _________________________ ID Number: ____________

Demographics:

1. How old are you?	Years: ________
2. Do you have medical insurance now?	Yes<br>No<br>I don't remember
3. If you have medical insurance, what type of insurance do you currently have?	☐ Public (SeNaSa)<br>☐ Private<br>☐ None
4. May I see your insurance card?	Yes<br>No<br>I don't have it<br>I would prefer not to show it
5. What type of employment do you currently have?	☐ Formal Employment<br>☐ Informal Employment<br>☐ Not employed
6. What is your highest level of education achieved?	☐ None<br>☐ Primary<br>☐ Secondary<br>☐ University
7. What was the first date of your last menstrual period?	Day/Month/Year: _________<br>Menopausal
8. Are you currently pregnant?	☐ Yes<br>☐ No

9. How many pregnancies have you had?	Total: ___________			
10. What is your nationality?	☐ Dominican<br>☐ Haitian<br>☐ Other: _________<br>☐ Prefer not to answer			
11. What is your native language	☐ Spanish<br>☐ Creole<br>☐ English<br>☐ Other: __________<br>☐ Prefer not to answer			
12. Did someone speak your native language during the birthing process for your children?	☐ Yes<br>☐ No			
13. What are the current ages and first names of your child(ren)? Identifying information will be destroyed after the interview.	Child 1: ___________	Child 2: _________	Child 3: ___________	Child 4: ___________
**Prenatal Care:**				
These questions will be asked for every pregnancy.<br>For the following questions, please answer them about your prenatal care experience with __________ (insert name of Child).	Child A:<br>________________	Child B:<br>________________	Child C:<br>________________	Child D:<br>_________________
14. For your pregnancy with ______ (insert name of Child here), did you receive prenatal care?	☐ Yes<br>☐ No	☐ Yes<br>☐ No	☐ Yes<br>☐ No	☐ Yes<br>☐ No
15. In your pregnancy with ____ (insert name of Child here), did you need medical insurance to get prenatal care?	Yes<br>No<br>I don't remember<br>I didn't have insurance	Yes<br>No<br>I don't remember<br>I didn't have insurance	Yes<br>No<br>I don't remember<br>I didn't have insurance	Yes<br>No<br>I don't remember<br>I didn't have insurance
16. What type of insurance did you have during your pregnancy with ____ (insert name of Child here)?	Private<br>Public<br>I don't remember<br>I don't have insurance	Private<br>Public<br>I don't remember<br>I don't have insurance	Private<br>Public<br>I don't remember<br>I don't have insurance	Private<br>Public<br>I don't remember<br>I don't have insurance
If the answer is **no**, skip to question **#27**<br>If the answer is **yes**, continue with question **#17**				

17. Where did you receive it?	☐ Public hospital<br>☐ Private hospital<br>☐ At home<br>☐ In my community<br>☐ Community Clinic<br>☐ Other: __________	☐ Public hospital<br>☐ Private hospital<br>☐ At home<br>☐ In my community<br>☐ Community Clinic<br>☐ Other: __________	☐ Public hospital<br>☐ Private hospital<br>☐ At home<br>☐ In my community<br>☐ Community Clinic<br>☐ Other: __________	☐ Public hospital<br>☐ Private hospital<br>☐ At home<br>☐ In my community<br>☐ Community Clinic<br>☐ Other: __________
18. Who was your main provider of prenatal care? (Choose one)	☐ Doctor<br>☐ Nurse<br>☐ Midwife<br>☐ Family Member<br>☐ Cooperadora<br>☐ ILAC Professional<br>☐ Other: __________	☐ Doctor<br>☐ Nurse<br>☐ Midwife<br>☐ Family Member<br>☐ Cooperadora<br>☐ ILAC Professional<br>☐ Other: __________	☐ Doctor<br>☐ Nurse<br>☐ Midwife<br>☐ Family Member<br>☐ Cooperadora<br>☐ ILAC Professional<br>☐ Other: __________	☐ Doctor<br>☐ Nurse<br>☐ Midwife<br>☐ Family Member<br>☐ Cooperadora<br>☐ ILAC Professional<br>☐ Other: __________
19. Approximately, how many prenatal visits did you have?	☐ 0<br>☐ 1-3<br>☐ 4-7<br>☐ 8+	☐ 0<br>☐ 1-3<br>☐ 4-7<br>☐ 8+	☐ 0<br>☐ 1-3<br>☐ 4-7<br>☐ 8+	☐ 0<br>☐ 1-3<br>☐ 4-7<br>☐ 8+
20. When in your pregnancy did your visits start?	☐ 0-3 months<br>☐ 4-6 months<br>☐ >7 months	☐ 0-3 months<br>☐ 4-6 months<br>☐ >7 months	☐ 0-3 months<br>☐ 4-6 months<br>☐ >7 months	☐ 0-3 months<br>☐ 4-6 months<br>☐ >7 months
21. Did anyone discuss feeding your child or lactation at your visits?	☐ Yes<br>☐ No	☐ Yes<br>☐ No	☐ Yes<br>☐ No	☐ Yes<br>☐ No
22. Did anyone discuss your birthing plan at your visits?	☐ Yes<br>☐ No	☐ Yes<br>☐ No	☐ Yes<br>☐ No	☐ Yes<br>☐ No
23. Did you plan for a vaginal or C-section?	☐ Vaginal<br>☐ C-Section	☐ Vaginal<br>☐ C-Section	☐ Vaginal<br>☐ C-Section	☐ Vaginal<br>☐ C-Section
24. At most times, how did you get to your visits?	☐ Car<br>☐ Walking<br>☐ Someone else's car<br>☐ Moto<br>☐ Public transport<br>☐ GuaGua<br>☐ Animal<br>☐ Other: __________	☐ Car<br>☐ Walking<br>☐ Someone else's car<br>☐ Moto<br>☐ Public transport<br>☐ GuaGua<br>☐ Animal<br>☐ Other: __________	☐ Car<br>☐ Walking<br>☐ Someone else's car<br>☐ Moto<br>☐ Public transport<br>☐ GuaGua<br>☐ Animal<br>☐ Other: __________	☐ Car<br>☐ Walking<br>☐ Someone else's car<br>☐ Moto<br>☐ Public transport<br>☐ GuaGua<br>☐ Animal<br>☐ Other: __________
25. Did anyone attend prenatal visits with you?	☐ Yes<br>☐ No	☐ Yes<br>☐ No	☐ Yes<br>☐ No	☐ Yes<br>☐ No
26. If yes, who came with you? (Choose all	☐ Midwife	☐ Midwife	☐ Midwife	☐ Midwife

that apply)	☐ Partner<br>☐ Parent(s)<br>☐ Friend(s)<br>☐ Other children<br>☐ Other: __________	☐ Partner<br>☐ Parent(s)<br>☐ Friend(s)<br>☐ Other children<br>☐ Other: __________	☐ Partner<br>☐ Parent(s)<br>☐ Friend(s)<br>☐ Other children<br>☐ Other: __________	☐ Partner<br>☐ Parent(s)<br>☐ Friend(s)<br>☐ Other children<br>☐ Other: __________

Repeat for all pregnancies from question #14 through #26 then continue to question #27

Delivery Method:

These questions will be asked for every pregnancy.<br>For the following questions, please answer them about your birth experience with __________(insert name of Child).	Child<br>______________	Child<br>______________	Child<br>______________	Child<br>______________
27. Did you need medical insurance to give birth?	Yes<br>No<br>I don't remember<br>I didn't have it	Yes<br>No<br>I don't remember<br>I didn't have it	Yes<br>No<br>I don't remember<br>I didn't have it	Yes<br>No<br>I don't remember<br>I didn't have it
28. If you needed insurance to give birth, what type of insurance did you have?	Private<br>Public<br>I don't remember<br>I didn't have insurance	Private<br>Public<br>I don't remember<br>I didn't have insurance	Private<br>Public<br>I don't remember<br>I didn't have insurance	Private<br>Public<br>I don't remember<br>I didn't have insurance
29. Where did you deliver? Specifically, which city or province?	City:<br>______________	City:<br>______________	City:<br>______________	City:<br>______________
30. What type of facility did you deliver in?	☐ Private hospital<br>☐ Public hospital<br>☐ Community Clinic<br>☐ Home<br>☐ Other: __________	☐ Private hospital<br>☐ Public hospital<br>☐ Community Clinic<br>☐ Home<br>☐ Other: __________	☐ Private hospital<br>☐ Public hospital<br>☐ Community Clinic<br>☐ Home<br>☐ Other: __________	☐ Private hospital<br>☐ Public hospital<br>☐ Community Clinic<br>☐ Home<br>☐ Other: __________
31. How far in minutes/hours did you have to travel to give birth?	Time:<br>______________	Time:<br>______________	Time:<br>______________	Time:<br>______________
32. What kind of delivery did you have?	☐ Vaginal<br>☐ C-Section	☐ Vaginal<br>☐ C-Section	☐ Vaginal<br>☐ C-Section	☐ Vaginal<br>☐ C-Section

If the answer is **vaginal**, proceed with questions **#33-35**
If the answer is **C-section**, proceed with questions **#36-40**

33. What motivated you to have a vaginal	☐ Provider preference	☐ Provider preference	☐ Provider preference	☐ Provider preference

delivery?	☐ Recovery Time<br>☐ Partner preference<br>☐ Perceived Effort<br>☐ Perceived Pain<br>☐ My preference<br>☐ Other: ___________	☐ Recovery Time<br>☐ Partner preference<br>☐ Perceived Effort<br>☐ Perceived Pain<br>☐ My preference<br>☐ Other: ___________	☐ Recovery Time<br>☐ Partner preference<br>☐ Perceived Effort<br>☐ Perceived Pain<br>☐ My preference<br>☐ Other: ___________	☐ Recovery Time<br>☐ Partner preference<br>☐ Perceived Effort<br>☐ Perceived Pain<br>☐ My preference<br>☐ Other: ___________
34. If answer to **#33 was recovery time**, how long did your recovery last in weeks?	Weeks: ______________	Weeks: ______________	Weeks: ______________	Weeks: ______________
35. Who was most influential in your decision to have a vaginal delivery?	☐ Myself<br>☐ Obstetrician<br>☐ Nurse<br>☐ Midwife<br>☐ Partner<br>☐ Parents<br>☐ Friends<br>☐ Course/education<br>☐ Internet<br>☐ Television<br>☐ Social Media<br>☐ Newspaper/Magazine/ Book/Journal<br>☐ Other: ___________	☐ Myself<br>☐ Obstetrician<br>☐ Nurse<br>☐ Midwife<br>☐ Partner<br>☐ Parents<br>☐ Friends<br>☐ Course/education<br>☐ Internet<br>☐ Television<br>☐ Social Media<br>☐ Newspaper/Magazine/ Book/Journal<br>☐ Other: ___________	☐ Myself<br>☐ Obstetrician<br>☐ Nurse<br>☐ Midwife<br>☐ Partner<br>☐ Parents<br>☐ Friends<br>☐ Course/education<br>☐ Internet<br>☐ Television<br>☐ Social Media<br>☐ Newspaper/Magazine/ Book/Journal<br>☐ Other: ___________	☐ Myself<br>☐ Obstetrician<br>☐ Nurse<br>☐ Midwife<br>☐ Partner<br>☐ Parents<br>☐ Friends<br>☐ Course/education<br>☐ Internet<br>☐ Television<br>☐ Social Media<br>☐ Newspaper/Magazine/ Book/Journal<br>☐ Other: ___________
36. Did you have an emergent C-section?	☐ Yes<br>☐ No	☐ Yes<br>☐ No	☐ Yes<br>☐ No	☐ Yes<br>☐ No
37. If **no for #36**, was your C-section planned?	☐ Yes<br>☐ No	☐ Yes<br>☐ No	☐ Yes<br>☐ No	☐ Yes<br>☐ No
38. What motivated your choice to have a C-section?	☐ Provider preference<br>☐ Recovery Time<br>☐ Partner preference<br>☐ Perceived Effort<br>☐ Perceived Pain<br>☐ My preference<br>☐ Other: ___________	☐ Provider preference<br>☐ Recovery Time<br>☐ Partner preference<br>☐ Perceived Effort<br>☐ Perceived Pain<br>☐ My preference<br>☐ Other: ___________	☐ Provider preference<br>☐ Recovery Time<br>☐ Partner preference<br>☐ Perceived Effort<br>☐ Perceived Pain<br>☐ My preference<br>☐ Other: ___________	☐ Provider preference<br>☐ Recovery Time<br>☐ Partner preference<br>☐ Perceived Effort<br>☐ Perceived Pain<br>☐ My preference<br>☐ Other: ___________
39. If answer to **#38 was recovery time**, how long did your recovery last in weeks?	Weeks: ______________	Weeks: ______________	Weeks: ______________	Weeks: ______________

40. Who was most influential in your decision to have a C-section?	☐ Myself<br>☐ Obstetrician<br>☐ Nurse<br>☐ Midwife<br>☐ Partner<br>☐ Parents<br>☐ Friends<br>☐ Course/education<br>☐ Internet<br>☐ Television<br>☐ Social Media<br>☐ Newspaper/Magazine/ Book/Journal<br>☐ Other: ___________	☐ Myself<br>☐ Obstetrician<br>☐ Nurse<br>☐ Midwife<br>☐ Partner<br>☐ Parents<br>☐ Friends<br>☐ Course/education<br>☐ Internet<br>☐ Television<br>☐ Social Media<br>☐ Newspaper/Magazine/ Book/Journal<br>☐ Other: ___________	☐ Myself<br>☐ Obstetrician<br>☐ Nurse<br>☐ Midwife<br>☐ Partner<br>☐ Parents<br>☐ Friends<br>☐ Course/education<br>☐ Internet<br>☐ Television<br>☐ Social Media<br>☐ Newspaper/Magazine/ Book/Journal<br>☐ Other: ___________	☐ Myself<br>☐ Obstetrician<br>☐ Nurse<br>☐ Midwife<br>☐ Partner<br>☐ Parents<br>☐ Friends<br>☐ Course/education<br>☐ Internet<br>☐ Television<br>☐ Social Media<br>☐ Newspaper/Magazine/ Book/Journal<br>☐ Other: ___________
41. Are you satisfied with your birth experience?	☐ Yes<br>☐ No	☐ Yes<br>☐ No	☐ Yes<br>☐ No	☐ Yes<br>☐ No
Repeat for all pregnancies from question #27 through #40 than continue on to question #42.				

Immediately Postpartum

These questions will be asked for every pregnancy<br>For the following questions, please answer them about your postpartum care experience with ___________ (insert name of Child here).	Child ________________	Child ________________	Child ________________	Child ________________
42. Did you have skin-to-skin contact immediately after birth?	☐ Yes<br>☐ No	☐ Yes<br>☐ No	☐ Yes<br>☐ No	☐ Yes<br>☐ No
43. Immediately after birth or after skin-to-skin contact was your baby taken to a different room?	☐ Yes<br>☐ No	☐ Yes<br>☐ No	☐ Yes<br>☐ No	☐ Yes<br>☐ No
If **yes**, go to question **#44**<br>If **no**, go to question **#45**				
44. How long were you separated?	☐ Less than an hour<br>☐ 1-2 hours<br>☐ >2 hours	☐ Less than an hour<br>☐ 1-2 hours<br>☐ >2 hours	☐ Less than an hour<br>☐ 1-2 hours<br>☐ >2 hours	☐ Less than an hour<br>☐ 1-2 hours<br>☐ >2 hours
45. Were you encouraged to breastfeed	☐ Yes	☐ Yes	☐ Yes	☐ Yes

immediately after the birth?	☐ No	☐ No	☐ No	☐ No
46. Was the baby's first meal breast milk or formula?	☐ Breast milk<br>☐ Formula	☐ Breast milk<br>☐ Formula	☐ Breast milk<br>☐ Formula	☐ Breast milk<br>☐ Formula
If **formula**, got to question **#47.**<br>If **breast milk**, begin the repeat at question #42 for remaining pregnancies or move on to question #42				
47. Who gave you the formula?	☐ Doctor<br>☐ Nurse<br>☐ Midwife<br>☐ Family<br>☐ Friends<br>☐ Self<br>☐ Other: ___________	☐ Doctor<br>☐ Nurse<br>☐ Midwife<br>☐ Family<br>☐ Friends<br>☐ Self<br>☐ Other: ___________	☐ Doctor<br>☐ Nurse<br>☐ Midwife<br>☐ Family<br>☐ Friends<br>☐ Self<br>☐ Other: ___________	☐ Doctor<br>☐ Nurse<br>☐ Midwife<br>☐ Family<br>☐ Friends<br>☐ Self<br>☐ Other: ___________
Repeat for all pregnancies from question #42 through #47 than continue on to question #48.				

**<u>Lactation:</u>**				
These questions will be asked for every pregnancy<br>For the following questions, please answer them about your postpartum care experience with ___________ (insert name of Child here).	Child<br>________________	Child<br>________________	Child<br>________________	Child<br>________________
48. Did you ever exclusively breastfeed, begin with formula feeding, or start with a combination of both?	☐ EBF<br>☐ Formula<br>☐ Combination	☐ EBF<br>☐ Formula<br>☐ Combination	☐ EBF<br>☐ Formula<br>☐ Combination	☐ EBF<br>☐ Formula<br>☐ Combination
If **EBF**, go to **#49**<br>If **Formula**, go to **#50**<br>If **Combination**, go to **#51**<br>After that question, move to #52				
49. How long did you exclusively breastfeed?	☐ Less than 6 months<br>☐ 6 months<br>☐ 7-12 months<br>☐ >12 months	☐ Less than 6 months<br>☐ 6 months<br>☐ 7-12 months<br>☐ >12 months	☐ Less than 6 months<br>☐ 6 months<br>☐ 7-12 months<br>☐ >12 months	☐ Less than 6 months<br>☐ 6 months<br>☐ 7-12 months<br>☐ >12 months
50. Was the formula easy to access?	☐ Yes<br>☐ No	☐ Yes<br>☐ No	☐ Yes<br>☐ No	☐ Yes<br>☐ No
51. Besides breast milk, what else did you	☐ Formula	☐ Formula	☐ Formula	☐ Formula

feed your infant?	☐ Animal/non-human milk<br>☐ Solid Food<br>☐ Other:_______	☐ Animal/non-human milk<br>☐ Solid Food<br>☐ Other:_______	☐ Animal/non-human milk<br>☐ Solid Food<br>☐ Other:_______	☐ Animal/non-human milk<br>☐ Solid Food<br>☐ Other:_______
52. Who was your primary influence in deciding how you fed your baby? (Choose one)	☐ Doctor<br>☐ Nurse<br>☐ Midwife<br>☐ Family<br>☐ Neighbor<br>☐ Social media<br>☐ Book<br>☐ Television<br>☐ Education<br>☐ Other: ___________	☐ Doctor<br>☐ Nurse<br>☐ Midwife<br>☐ Family<br>☐ Neighbor<br>☐ Social media<br>☐ Book<br>☐ Television<br>☐ Education<br>☐ Other: ___________	☐ Doctor<br>☐ Nurse<br>☐ Midwife<br>☐ Family<br>☐ Neighbor<br>☐ Social media<br>☐ Book<br>☐ Television<br>☐ Education<br>☐ Other: ___________	☐ Doctor<br>☐ Nurse<br>☐ Midwife<br>☐ Family<br>☐ Neighbor<br>☐ Social media<br>☐ Book<br>☐ Television<br>☐ Education<br>☐ Other: ___________
If the participant fed via **EBF or combo**, use **#53A**<br>If the participant only fed via **formula**, use **#53B**				
53. A. Did you feel supported in breastfeeding?	☐ Yes<br>☐ No	☐ Yes<br>☐ No	☐ Yes<br>☐ No	☐ Yes<br>☐ No
53 B. If you had chosen to breastfeed, do you think you would have been supported in that choice?	☐ Yes<br>☐ No	☐ Yes<br>☐ No	☐ Yes<br>☐ No	☐ Yes<br>☐ No
If the participant fed via **EBF or combo**, use **#54A.**<br>If the participant only fed via **formula**, use **#54B.**				
54. A. If you have a partner, was your partner supportive of breastfeeding?	☐ Yes<br>☐ No<br>☐ No partner	☐ Yes<br>☐ No<br>☐ No partner	☐ Yes<br>☐ No<br>☐ No partner	☐ Yes<br>☐ No<br>☐ No partner
54. B. If you had chosen to breastfeed, and if you have a partner, do you think your partner would have been supportive of that choice?	☐ Yes<br>☐ No<br>☐ No partner	☐ Yes<br>☐ No<br>☐ No partner	☐ Yes<br>☐ No<br>☐ No partner	☐ Yes<br>☐ No<br>☐ No partner
If the participant fed via **EBF or combo**, use **#55A**<br>If the participant only fed via **formula,** use **#55B**				
55. A. If you have other family members,	☐ Yes	☐ Yes	☐ Yes	☐ Yes

were your other family members supportive of breastfeeding?	☐ No<br>☐ No other family members	☐ No<br>☐ No other family members	☐ No<br>☐ No other family members	☐ No<br>☐ No other family members
55 B. If you had chosen to breastfeed, and if you have other family members, do you think any other family members would have been supportive of your choice to breastfeed?	☐ Yes<br>☐ No<br>☐ No other family members	☐ Yes<br>☐ No<br>☐ No other family members	☐ Yes<br>☐ No<br>☐ No other family members	☐ Yes<br>☐ No<br>☐ No other family members
Repeat for all pregnancies from question #48 through #55 then continue on to question #56.				

**These questions will be asked only one time**	
56. Do you think that your breast milk contains enough nutrients on its own to feed your baby?	☐ Yes<br>☐ No
57. Do you think that you produce enough breast milk to exclusively breastfeed without need of supplementation?	☐ Yes<br>☐ No
58. Is there anything specific that would help you breastfeed or make you more comfortable breastfeeding? (Choose one)	☐ More educational resources<br>☐ Increased support from medical professionals immediately after birth<br>☐ Increased family support<br>☐ Increased peer support<br>☐ Nothing additional required<br>☐ Other: ___________

**Sexual Health Education:**				
These questions will be asked for every pregnancy<br>For the following questions, please answer them about your prenatal care experience with ___________ (insert name of Child here).	Child ________________	Child ________________	Child ________________	Child ________________
59. Did you get tested for STI's during prenatal care?	☐ Yes<br>☐ No<br>☐ Do not remember	☐ Yes<br>☐ No<br>☐ Do not remember	☐ Yes<br>☐ No<br>☐ Do not remember	☐ Yes<br>☐ No<br>☐ Do not remember
60. Did you receive education on sexually transmitted infections (STI's) during	☐ Yes<br>☐ No	☐ Yes<br>☐ No	☐ Yes<br>☐ No	☐ Yes<br>☐ No

prenatal care?	☐ Do not remember	☐ Do not remember	☐ Do not remember	☐ Do not remember
If **yes or do not remember**, proceed with to **#61.** If **no**, go to **#66**				
61. Were you educated on gonorrhea during prenatal care?	☐ Yes<br>☐ No<br>☐ Do not remember	☐ Yes<br>☐ No<br>☐ Do not remember	☐ Yes<br>☐ No<br>☐ Do not remember	☐ Yes<br>☐ No<br>☐ Do not remember
62. Were you educated on chlamydia during prenatal care?	☐ Yes<br>☐ No<br>☐ Do not remember	☐ Yes<br>☐ No<br>☐ Do not remember	☐ Yes<br>☐ No<br>☐ Do not remember	☐ Yes<br>☐ No<br>☐ Do not remember
63. Were you educated on syphilis during prenatal care?	☐ Yes<br>☐ No<br>☐ Do not remember	☐ Yes<br>☐ No<br>☐ Do not remember	☐ Yes<br>☐ No<br>☐ Do not remember	☐ Yes<br>☐ No<br>☐ Do not remember
64. Were you educated on HIV during prenatal care?	☐ Yes<br>☐ No<br>☐ Do not remember	☐ Yes<br>☐ No<br>☐ Do not remember	☐ Yes<br>☐ No<br>☐ Do not remember	☐ Yes<br>☐ No<br>☐ Do not remember
65. Were you educated on Hepatitis B during prenatal care?	☐ Yes<br>☐ No<br>☐ Do not remember	☐ Yes<br>☐ No<br>☐ Do not remember	☐ Yes<br>☐ No<br>☐ Do not remember	☐ Yes<br>☐ No<br>☐ Do not remember
66. Were you diagnosed with a STI during pregnancy?	☐ Yes<br>☐ No<br>☐ Do not remember	☐ Yes<br>☐ No<br>☐ Do not remember	☐ Yes<br>☐ No<br>☐ Do not remember	☐ Yes<br>☐ No<br>☐ Do not remember
If **yes**, proceed to **#67.**<br>If **no or do not remember,** skip to **#69.**				
67. Which STI(s)? (Choose all that apply)	☐ Gonorrhea<br>☐ Chlamydia<br>☐ Syphilis<br>☐ HIV<br>☐ Hepatitis B<br>☐ Other: ___________	☐ Gonorrhea<br>☐ Chlamydia<br>☐ Syphilis<br>☐ HIV<br>☐ Hepatitis B<br>☐ Other: ___________	☐ Gonorrhea<br>☐ Chlamydia<br>☐ Syphilis<br>☐ HIV<br>☐ Hepatitis B<br>☐ Other: ___________	☐ Gonorrhea<br>☐ Chlamydia<br>☐ Syphilis<br>☐ HIV<br>☐ Hepatitis B<br>☐ Other: ___________
68. Did you receive treatment for it?	☐ Yes<br>☐ No<br>☐ Do not remember	☐ Yes<br>☐ No<br>☐ Do not remember	☐ Yes<br>☐ No<br>☐ Do not remember	☐ Yes<br>☐ No<br>☐ Do not remember
69. Did you use condoms during pregnancy?	☐ Yes<br>☐ No<br>☐ Do not remember	☐ Yes<br>☐ No<br>☐ Do not remember	☐ Yes<br>☐ No<br>☐ Do not remember	☐ Yes<br>☐ No<br>☐ Do not remember

If **yes**, proceed with **#70**
If **no or do not remember**, skip to **#72**

70. How often did you use condoms during pregnancy?	☐ Always<br>☐ Sometimes<br>☐ Rarely	☐ Always<br>☐ Sometimes<br>☐ Rarely	☐ Always<br>☐ Sometimes<br>☐ Rarely	☐ Always<br>☐ Sometimes<br>☐ Rarely
71. Why did you use them?	☐ STI Prevention<br>☐ Multiple Partners<br>☐ New Partner<br>☐ Positive STI Diagnosis<br>☐ Other: ___________	☐ STI Prevention<br>☐ Multiple Partners<br>☐ New Partner<br>☐ Positive STI Diagnosis<br>☐ Other: ___________	☐ STI Prevention<br>☐ Multiple Partners<br>☐ New Partner<br>☐ Positive STI Diagnosis<br>☐ Other: ___________	☐ STI Prevention<br>☐ Multiple Partners<br>☐ New Partner<br>☐ Positive STI Diagnosis<br>☐ Other: ___________
72. Did you receive education on family planning during prenatal care?	☐ Yes<br>☐ No<br>☐ Do not remember	☐ Yes<br>☐ No<br>☐ Do not remember	☐ Yes<br>☐ No<br>☐ Do not remember	☐ Yes<br>☐ No<br>☐ Do not remember

Repeat for all pregnancies from question #59 through #72 than continue on to question #73

These questions will be asked only one time

73. Do you currently desire to have more children?	☐ Yes<br>☐ No
74. Are you or your partner(s) currently doing something or using any method to delay or avoid getting pregnant?	☐ Yes<br>☐ No

If **yes**, proceed with question **#75.**
If **no**, skip to question **#76.**

75. Which method(s) are you using? (Choose all that apply)	☐ Barrier<br>☐ Condom<br>☐ Pill<br>☐ Injection<br>☐ Arm implant<br>☐ Tubal ligation<br>☐ IUD<br>☐ Natural Family Planning/Standard Days<br>☐ Vasectomy<br>☐ Other: ___________

76. Whether you use a family planning method or not, is it easy to access a family planning method?	Yes<br>No

5
