# Supplementary material for: Postpartum family planning education during prenatal care: a cross-sectional study using descriptive statistics in Barrero, Dominican Republic
Source: Front Glob Womens Health. 2026 Jul 22;7:1804322. doi: 10.3389/fgwh.2026.1804322 (PMC13437963; doi:10.3389/fgwh.2026.1804322)
Supplement: Supplementary file 3 [file Datasheet3.pdf]

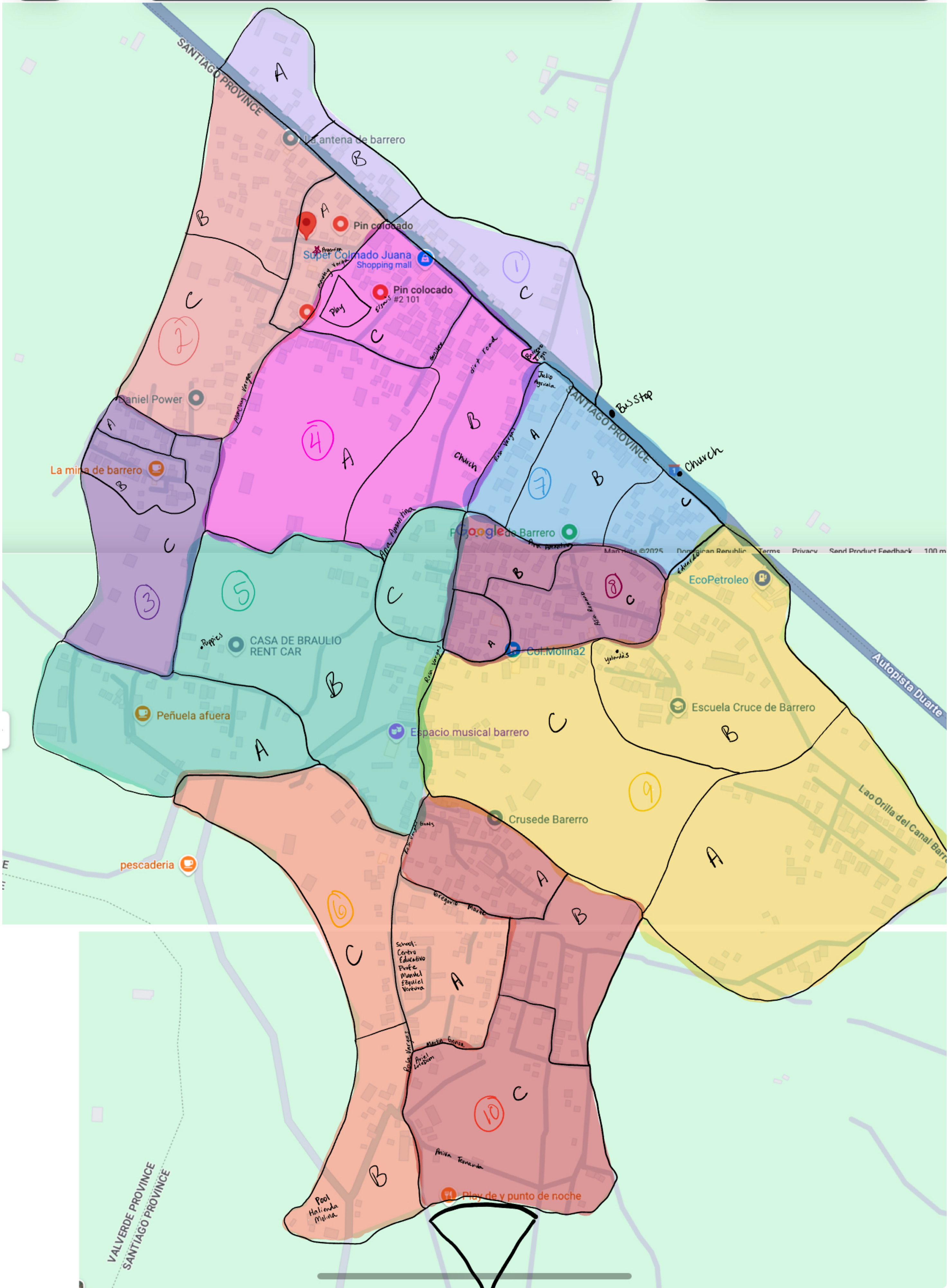

SANTIAGO PROVINCE
La antena de barrero
Pin colocado
Super Colmado Juana
Shopping mall
Pin colocado
#2 101
Play
dirt road
Church
Daniel Power
La mina de barrero
Bus Stop
Church
SANTIAGO PROVINCE
CASA DE BRAULIO
RENT CAR
Peñuela afuera
Espacio musical barrero
EcoPetroleo
Col.Molina2
Escuela Cruce de Barrero
Autopista Duarte
Crusede Barerro
pescaderia
School: Centro Educativo Profe Manuel Ezequiel Ventura
Pool Hacienda Molina
Play de y punto de noche
VALVERDE PROVINCE
SANTIAGO PROVINCE
Map data ©2025
Terms
Privacy
Send Product Feedback
